# Editorial

**Published:** 1904-11-15

**Authors:** 


### EDITORIAL.

THE editor of the *International Journal*, reviewing the International Dental Congress, criticises sharply the most obvious shortcomings of that meeting, and we are not disposed to controvert his conclusions. But he raises the question as to the section method of presenting the program of such large conventions. Here, too, we are in agreement, but like "Boss Tweed," the celebrated Tammany leader, who, when confronted with his palpable misuse of a sacred public trust, acknowledged the facts, but simply said, "What are you going to do about it?" So long as the programs of our National meetings, for instance, are made up as they are by voluntary contributions, it will be impracticable to give each paper a hearing and proper consideration in the general sessions. Conditions are against any satisfactory adjustment of this problem. If the program committee attempts to reject a paper it will have troubles in plenty. If it takes on and attempts to provide a hearing for all the papers offered, the discussions must be curtailed or eliminated entirely, and more troubles result. If the meeting is prolonged over three days the members get tired and go home or neglect the meetings for local sight-seeing and other diversions. If the committee decides to have fewer papers and selects the subjects and authors who will adequately represent them, some one is certain to be offended if not invited, and complains that politics is running the Association and he will have nothing more to do with it. We might go on and give other excuses that have been made for failures in the manner of conducting our great gatherings. The truth of the matter seems to be that the large majority of our profession are like the man from Missouri, "they want to be shown things." There is no trouble to get a crowd when a big clinic is assured, and there is no trouble about getting the attendance when there is something to be learned that appeals to the every-day practical work of the practitioner. Dentists don't read, except the newspapers, and they can not concentrate their interest on long tedious essays, which have no special application to their every-day work. Since this is a fact, which we of course regret, would it not be the part of wisdom to act upon it, and prepare our programs with more care in the future? Have better and fewer papers, and more and better clinics. The method of giving clinics at present is about as unsatisfactory as anything can be. Can't we change this by study? A pointer worth thinking of was the presentation of cavity preparation and gold filling by about twenty operators, under the direction of Dr. Wedelstadt, at the International Congress. They illustrated an ideal and they did it well, and taught successfully the idea Dr. Wedelstadt has been for years writing about. Hereafter in our National Society we shall have but three sections, two more than are desirable perhaps, but let the chairmen of these sections make up a program of what kind of papers they want, and how many they can give proper consideration, and then select the men who are to write, and give them time to prepare papers that will be worth hearing, and which shall have such consdieration that an author will be willin gto labor to present his subject worthily for the honor conferred. This would result in much good. The meetings would have enough of value to justify the expense of time and money involved in attending them, and the results would include the best contributions of the profession. All this could be accomplished without changing the constitution or offending persons who want to come in at the last hour with a paper which has been constructed on the journey to the meeting, or, as we once knew of a prominent contributor doing, after he had reached the meeting with the aid of a drug clerk and a dispensatory. If we must have the section plan, make it as acceptable as possible.

